# Improvement in Depressive Symptoms Is Associated with Reduced Oxidative Damage and Inflammatory Response in Type 2 Diabetic Patients with Subsyndromal Depression: The Results of a Randomized Controlled Trial Comparing Psychoeducation, Physical Exercise, and Enhanced Treatment as Usual

**DOI:** 10.1155/2015/210406

**Published:** 2015-08-10

**Authors:** Marijana Vučić Lovrenčić, Mirjana Pibernik-Okanović, Mario Šekerija, Manja Prašek, Dea Ajduković, Jadranka Kos, Norbert Hermanns

**Affiliations:** ^1^Department of Laboratory Medicine, Merkur University Hospital, Zajčeva 19, 10000 Zagreb, Croatia; ^2^Vuk Vrhovac University Clinic for Diabetes, Merkur University Hospital, Zajčeva 19, 10000 Zagreb, Croatia; ^3^Croatian Institute of Public Health, Rockefellerova 7, 10000 Zagreb, Croatia; ^4^Forschungsinstitut Diabetes-Akademie Bad Mergentheim (FIDAM GmbH), Theodor Klotzbücher Strasse 12, 97980 Bad Mergentheim, Germany

## Abstract

*Aims*. To examine one-year changes in oxidative damage and inflammation level in type 2 diabetic patients undergoing behavioral treatment for subsyndromal depression. *Materials and Methods*. A randomized controlled comparison of psychoeducation (A), physical exercise (B), and enhanced treatment as usual (C) was performed in 209 eligible subjects in a tertiary diabetes care setting. Depressive symptoms (primary outcome) and selected biomarkers of oxidative damage and inflammation (secondary outcomes) were assessed at baseline and six- and twelve-month follow-up. *Results*. Out of the 74, 67, and 68 patients randomised into groups A, B, and C, respectively, 201 completed the interventions, and 179 were analysed. Participants in all three groups equally improved in depressive symptoms from baseline to one-year follow-up (repeated measures ANOVA; *F* = 12.51, *p* < 0.0001, *η* ^2^ = 0.07). Urinary 8-oxo-deoxyguanosine (u-8-oxodG) decreased (*F* = 10.66, *p* < 0.0001, *η* ^2^ = 0.06), as did sialic acid and leukocytes (*F* = 84.57, *η* ^2^ = 0.32 and *F* = 12.61, *η* ^2^ = 0.07, resp.; *p* < 0.0001), while uric acid increased (*F* = 12.53, *p* < 0.0001, *η* ^2^ = 0.07) in all subjects during one year. Improvement of depressive symptoms at 6 months significantly predicted one-year reduction in u-8-oxodG (*β* = 0.15, *p* = 0.044). *Conclusion*. Simple behavioral interventions are capable not only of alleviating depressive symptoms, but also of reducing the intensity of damaging oxidative/inflammatory processes in type 2 diabetic patients with subsyndromal depression. This trial is registered with ISRCTN05673017.

## 1. Introduction

Increased prevalence of elevated depressive symptoms in patients suffering from diabetes is well documented [1], as are their association with suboptimal metabolic control, diabetes complications, and mortality [2–6]. An increased incidence of adverse health outcomes was observed not only in patients with the established diagnosis of depression but also in those suffering subthreshold depressive symptoms [7], indicating that even mild depression is clinically relevant. Feasible and effective interventions that might provide benefits to both physical and mental health are appraised in order to optimize health outcomes in this vulnerable patient group [8, 9].

Recently, research in this area has been focused on biochemical markers reflecting common pathophysiological processes of insulin resistance, inflammation, and oxidative damage assumed to be intertwined in both diabetes and depression. Psychosocial stressors, inappropriate diet, sedentary lifestyle, obesity, and increased intestinal permeability have been proposed as worthwhile intervention targets for treatment or prevention of both diseases [10–12]. Inflammation was found to be associated with a combination of type 2 diabetes and depressive symptoms compared with depression alone and diabetes without depression [13]. Further investigation in these associations was stressed necessary for better understanding of biological pathways underlying both diabetes and depressive symptoms. Hypothesizing that depressive symptom scores in newly diagnosed type 2 diabetes patients are associated with higher concentrations of inflammatory markers, Laake et al. explored a large cohort of newly diagnosed type 2 diabetic patients. Their findings indicated that increased inflammation may be involved in the pathogenesis of depressive symptoms in type 2 diabetes, thus contributing to the increased risk of complications and mortality in this group [14]. Transient increases in systematic inflammation have been observed in response to acute psychosocial stress, with larger responses among individuals reporting adverse psychosocial states or conditions such as depression and low self-esteem [15]. Also, a hypothesis on the involvement of inflammatory as well as oxidative and nitrosative stress pathways in the pathogenesis of depression has received much attention [16]. Association between depression and oxidative damage due to both increased reactive oxygen species (ROS) production and decreased antioxidant capacity has been clearly evidenced in a recent meta-analysis, suggesting that oxidative stress might provide a plausible biochemical link between depression and unfavorable health outcomes [17]. The most likely generator of oxidative stress in depression is considered to be a chronic, low-grade inflammation resulting from activation of cellular immunity able to induce an immune-mediated damage to the cells. Diabetic patients are particularly vulnerable to these detrimental effects, since hyperglycemia* per se* promotes excessive production of ROS and depletion of antioxidant defence capacity, resulting in oxidative damage, that is, at least in part, presumably responsible for development of micro- and macrovascular diabetic complications [18].

In spite of the growing evidence that inflammation is associated with type 2 diabetes and depressive symptoms, research on the interventions focusing on the interaction between depressive symptoms and inflammatory response is still rare. Findings obtained in general population have shown that inflammatory markers were higher in patients suffering major depressive disorders than in those without depression and that they are significantly reduced during cognitive-behavioral therapy [19]. Whether improving depressive symptoms may lead to reduction in increased inflammation and oxidative damage in individuals suffering from diabetes has been insufficiently studied. Although a recent report has not observed significant influence of effective cognitive-behavioral intervention on some inflammatory variables in type 2 diabetic patients with poor glucoregulation and subclinical depression [20], the relationships between diabetes, oxidative stress, low-grade inflammation, and different levels of depressive symptoms remain unclear.

In this study, being a part of a broader RCT comparing the effects of treating subsyndromal depression, we aimed to examine one-year changes in selected biomarkers of oxidative stress and inflammation in type 2 diabetic patients treated for elevated depressive symptoms that do not reach criteria for clinical depression. Depressive symptoms were primary, while biomarkers of inflammation (leukocytes, hs-CRP, adiponectin, and sialic acid), oxidative damage (urinary 8-oxo-deoxyguanosine), and endogenous antioxidant capacity (uric acid) were secondary study outcomes.

## 2. Methods

A postal screening for depressive symptoms relying on the short form Patient Health Questionnaire-Depression (PHQ-2) [21] was used to recruit potential study participants. The invited patients (*n* = 4858) were identified in the patient registry based on inclusion criteria of having type 2 diabetes for at least one year, living in Zagreb, and attending at least one medical check-up during the previous year. Out of 1740 responders (36%), 763 did not report depressive symptoms and 205 expressed no need for receiving professional help in reported mood-related difficulties. After excluding patients with clinical depression and/or psychiatric treatment (*n* = 134), those with severe physical limitations (*n* = 94), and those with both physical and psychiatric limitations/illnesses (*n* = 55), or other obstacles for participation (*n* = 124), 365 patients were found to be eligible. Eligibility was determined in a phone interview inquiring about personal, disease-related, and sociodemographic data. A presence or absence of clinical depression was determined by phone-administered structured clinical interview for the DSM-IV Axis I disorders. Out of the 365 eligible patients, 265 agreed to participate; among the remaining 100, 58 refused to join the groups and 42 were unreachable after the first contact. Two hundred and nine patients, who gave their informed consent, were randomised to study groups and completed baseline assessments. A computer-generated algorithm stratified by gender was used to allocate patients to the groups and to harmonise the number of female and male participants in each group [22]. Research assistants were responsible for running computer-generated assignments of the participants to the study arms and conducting the enrolment. No deviations from computer-generated assignments occurred with exception of one patient who did not accept the proposed group when coming to the baseline assessment. This patient was not included in the study but suggested to choose between other sources of professional help.

**Figure 1:**
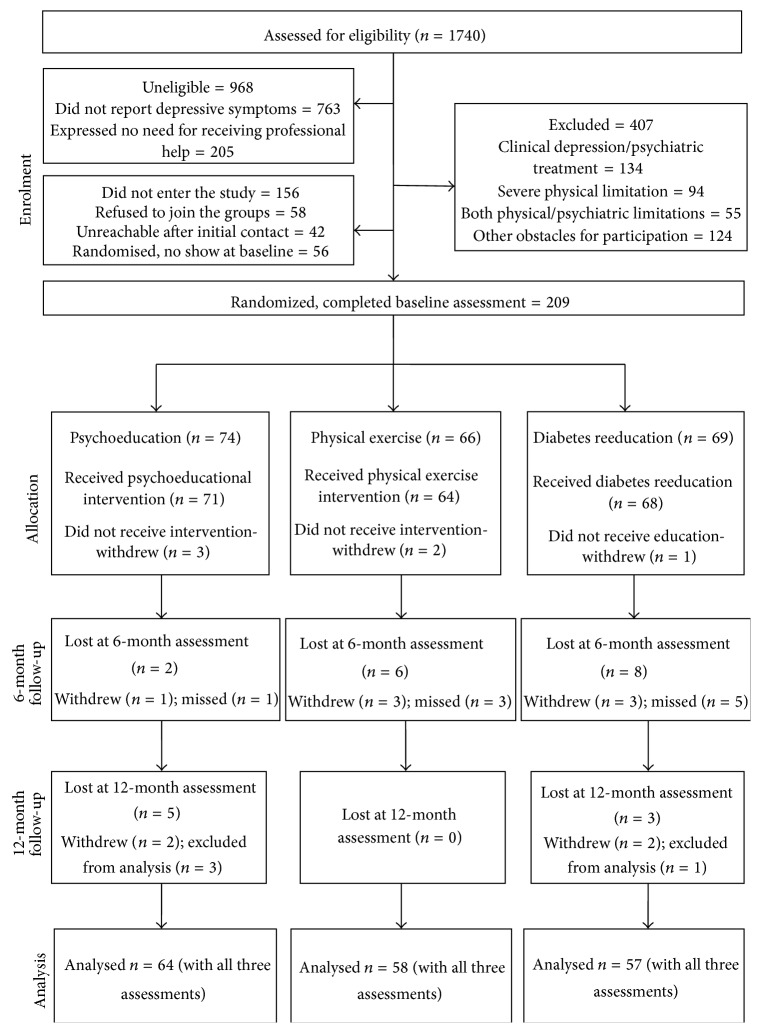
Patients' flow diagram.

Patients' flow is shown in Figure 1.

Primary study outcomes were depressive symptoms and secondary outcomes were endogenous pro- and anti-inflammatory markers (leukocytes, hs-CRP, sialic acid, and adiponectin), serum uric acid as an indicator of antioxidant capacity, and an overall measure of oxidative damage to DNA (urinary 8-oxo-deoxyguanosine). Sample size calculation was based on the absolute change in depressive symptoms as measured by the CES-D questionnaire. An improvement of 0.5 standard deviations was considered clinically relevant [23]. With alpha = 0.05, samples of *N* = 59 per group were shown to be needed to have 80% power in demonstrating statistically significant differences in depressive symptoms. A more detailed study procedure was described elsewhere [24].

The study was carried out at the Vuk Vrhovac University Clinic for Diabetes—Reference Centre for Diabetes Mellitus in Zagreb, Croatia. Laboratory analyses were performed at the Department of Laboratory Medicine, Merkur University Hospital, accredited according to the ISO 15189:2012 “medical laboratories—requirements for quality and competence” standard.

### 2.1. The Intervention Arms: Psychoeducation, Physical Exercise, and Enhanced Treatment as Usual

Two intervention programs—psychoeducation and physical exercise—and enhanced treatment as usual consisting of brief diabetes reeducation were offered to patients reporting elevated depressive symptoms and a need for receiving professional help in mood-related difficulties. The intervention programs were delivered in small groups attending six 90-minute weekly sessions. One 90-minute intervention session—enhanced treatment as usual—was offered to the control study participants because leaving these patients without any treatment was considered ethically unacceptable.

The interventions were considered to have a very low potential for causing harm. Nevertheless, patient information, being a part of the written consent, clearly stated that the participants can discontinue intervention at any time, particularly if they would perceive that their symptoms have been worsened. Qualified professionals involved in treatment carefully monitored patient's condition and routine laboratory results in order to provide a timely referral to physician's care, if needed. In addition, patients allocated to physical exercise underwent ECG and, if indicated, an ergometric test before the study. Exercise intensity was measured by a heart rate monitor and maintained in a light-to-medium intensity range, whereas blood glucose and blood pressure were measured before and after each session.

A summary of the programs' objectives, methods of delivery, and specific features are presented in Table 1.

### 2.2. Assessments

Patient demographic variables included age, sex, education, family status, employment, and economic status. Diabetes-related variables included years of diabetes duration and diabetes treatment with or without insulin.

Screening for mood difficulties was carried out using the adapted Patient Health Questionnaire-2 [21]. An additional question inquiring into the patients' need for help with these difficulties was added, as it has proven to improve the instrument's validity [25].

The presence or absence of clinical depression was determined by phone-administered structured clinical interview for the DSM-IV Axis 1 disorders, as this method has been shown to be comparable to face-to-face interview [26].

Depressive symptoms were measured by the* Center for Epidemiological Studies Depression* (CES-D)* Scale*, a 20-item self-report instrument [27]. The total score of the scale ranges from 0 to 60 points, with higher scores indicating higher depressive symptoms. The reliability of the scale at baseline was Cronbach alpha = 0.83.

#### 2.2.1. Laboratory Assessments

HbA_1c_ was measured by an NGSP-certified automated immunoturbidimetric method (Tina-quant HbA_1c_, Roche Diagnostics, USA) with a total imprecision (expressed as CV) <1.5% and dual reporting of the results (NGSP/DCCT aligned % and IFCC-aligned mmol/mol).

Leukocyte count was determined in K3EDTA-whole blood samples with ADVIA120 blood cell counter (Siemens Diagnostic Solutions, USA).

Serum uric acid and hs-CRP concentrations were measured by the automated enzymatic and immunoturbidimetric assays, respectively (AU680, Beckman Coulter, USA).

Total serum sialic acid (TSA) was determined by a colorimetric procedure, as described previously [28]. Serum adiponectin was measured by a commercially available sandwich ELISA procedure (Adiponectin Human ELISA, high sensitive, Biovendor, CZ) with declared detection limit of 0.47 *μ*g/L.

Urinary 8-oxo-deoxyguanosine (u-8-oxodG) was measured by the competitive ELISA method (New 8-OHdG Check, Japan Institute for the Control of Ageing, JaiCA, Shizuoka, Japan), with declared assay-range of 0.5–200 *μ*g/L and improved specificity [29]. On the day of analysis, frozen urine samples were thawed in a water-bath (37°C), vortexed, centrifuged (10 min, 3000 rpm), and u-8-oxodG assayed immediately according to the manufacturer's instructions. Urinary creatinine was measured in the same samples with a compensated colorimetric Jaffe procedure (AU680 Analyser, Beckman Coulter, Brea, USA). Results were normalized to the creatinine concentration by the formula u-8-oxodG/creatinine (*μ*g/mmol).

Variables indicating a level of depressive symptoms and biomarkers of oxidative damage and inflammation were measured at baseline and after six- and twelve-month follow-up periods. HbA_1c_, leukocytes, serum hs-CRP, and uric acid were assayed in fresh samples obtained in the morning after an overnight fast. Samples for sialic acid, adiponectin, and u-8-oxodG measurements were frozen (−70°C) until analyzed. The samples from each patient were analyzed with ELISAs at the same microplate in order to avoid possible between-series variability.

### 2.3. Statistical Methods

Data are presented as means (SD), unless otherwise indicated. Due to the nonparametric distribution hs-CRP results were log-transformed prior to the analysis. One-way ANOVA and chi squares were used to determine baseline differences across the three treatment arms and examine differences between continuing participants and dropouts.

Data were analyzed per protocol after exclusion of participants who withdrew, did not attend all three follow-up meetings, initiated psychopharmacological treatment, or died during the study period; these results are presented throughout the paper. Intention-to-treat (ITT) analysis was performed to validate these results; all participants who were randomized were analyzed despite deviations from the protocol. To perform ITT, missing data were imputed using the baseline observation carried forward approach.

Repeated measures ANOVA was used to test for changes in outcome variables across time and between the groups. Cohen's *η* ^2^ coefficients ranging from 0.01 to 0.05 were regarded as small, from 0.06 to 0.12 moderate, and ≥0.13 large.

Regression analysis was employed to test directional relations between depressive symptoms and reduction in inflammation and oxidative stress.

A *p* value of <0.05 was considered significant in all analyses.

Statistical analyses were performed by using the SPSS 17 (SPSS Inc., Chicago, IL, USA).

### 2.4. Ethical Aspects

The study was approved by the Vuk Vrhovac University Clinic for Diabetes Ethics Committee on June 30, 2010. A written informed consent was obtained from all the participants.

## 3. Results

Among the 1740 patients assessed for eligibility, 365 were considered eligible and 265 agreed to participate in the interventions (72%). Of these 209 completed baseline laboratory and psychological assessments (79%), with 74 randomized to psychoeducational intervention, 66 to physical exercise, and 69 to diabetes reeducation. Attrition rate was 3% during the interventions (6 of 209 patients), 3% from baseline to six months (7 of 203 patients), and 2% from 6-month to 12-month follow-up period (4 of 196 patients). Of the seven dropouts who completed the intervention but missed both follow-up assessments, two missed the follow-up appointments due to health problems, four were unwilling to come, and one patient died. No harmful incidents were recorded throughout the interventions. Four patients were excluded from per-protocol analyses due to the initiation of pharmacological therapy. No differences between the participants and dropouts across the three study groups were observed.

Table 2 presents demographic and diabetes-related characteristics of patients randomized to one of the three groups and a baseline level of depressive symptoms as self-assessed by the CES-D questionnaire. No between-group differences regarding demographic, diabetes-related, and psychological variables were found.

The effects of interventions on depressive symptoms and biomarkers of oxidative damage and inflammation, as well as glucoregulation (HbA_1c_) and BMI, based on repeated measures ANOVA are given in Table 3. ITT analyses showed a comparable pattern of results of all analyses in terms of statistical significance and effect size and are not presented in the text.

### 3.1. Changes in Depressive Symptoms

No significant between-group differences over time were obtained with respect to depressive symptoms (*F* = 0.61, *p* = 0.656), indicating comparable efficacy of psychoeducation, physical exercise, and enhanced treatment as usual on the primary study outcome.

Significant reductions in depressive symptoms occurred across all three study groups from baseline to 12 months (*F* = 12.51, *p* < 0.001, *η* ^2^ = 0.7). The size of the time effect indicated a moderate effect of the three treatments on self-assessed depressive symptoms.

### 3.2. Changes in Biomarkers of Oxidative Damage and Inflammation

Total leukocytes significantly decreased in all three groups (*F* = 12.61, *p* < 0.0001, *η* ^2^ = 0.07) as did u-8-oxodG (*F* = 10.66, *p* < 0.0001, *η* ^2^ = 0.06) and sialic acid (*F* = 84.57, *p* < 0.0001, *η* ^2^ = 0.32). The latter showed small but statistically significant time *x* group effect (*F* = 3.40, *p* = 0.01, *η* ^2^ = 0.04). However, after adjusting the results for multiple comparisons by Bonferroni test, it did not remain significant. Uric acid increased significantly over one year in all three groups (*F* = 13.39, *p* < 0.0001, *η* ^2^ = 0.07). The effects of the interventions on sialic acid can be regarded as large (0.32) and on leukocytes, u-8-oxodG, and uric acid as moderate (0.07, 0.06, and 0.07, resp.).

No significant changes were registered with respect to adiponectin and hs-CRP.

Neither HbA_1c_ nor BMI were significantly changed in any of the study groups from baseline to 12 months.

No between-group effects were obtained with respect to one-year changes in biomarkers of inflammation and oxidative damage (all *p* > 0.05).

### 3.3. Testing for Mediation

The results obtained by using regression analyses indicated that improvement in depressive symptoms at six months predicted reductions in u-8-oxodG at twelve-month follow-up (*β* = 0.15, *p* = 0.044), while the results for sialic and uric acid were close to statistical significance (*p* = 0.069 and *p* = 0.070, resp.) (results not shown in tables).

## 4. Discussion

This randomized interventional study attempted to evaluate the effect of two behavioral interventions: psychoeducation and physical exercise, as compared to enhanced treatment as usual, on depressive symptoms and the intensity of oxidative/inflammatory processes in diabetic patients suffering from subsyndromal depression. The results indicate protracted favorable effects of either intervention on lowering depressive symptoms while reducing overall oxidative damage and cellular inflammatory response, as well as improving antioxidant capacity.

In all three study arms a comparable and clinically meaningful improvement in depressive symptoms was obtained. The intervention-independent results regarding depression-related outcomes confirm our preliminary report indicating that treatment as usual was equally effective as psychoeducation in reducing depressive symptoms and improving metabolic control in type 2 diabetic patients with subsyndromal depression [30]. Also, no between-group differences were found within a randomized controlled comparison of psychoeducation, physical exercise, and control arm consisting of brief diabetes reeducation [31]. The absence of a significant treatment effect suggested that patterns of change might be similar in all treatment groups. Hypothetically, addressing patients' emotional needs and their diabetes-related distress which were common in all three approaches may indicate a possible pathway of change. Similar findings were obtained in a study by Fisher et al. [32] which demonstrated that clinically reasonable improvements in emotional symptoms may be stimulated by even minimal behavioral interventions.

In this study, a significant decrease in overall oxidative damage was observed from basal to one-year posttreatment follow-up by measuring a recognized biomarker of oxidative damage to DNA—urinary 8-oxodG. Urinary excretion of 8-oxodG reflects the average rate of nucleic acid guanine oxidation and can be regarded as a reliable marker of general intracellular oxidative stress [33]. Research on the u-8-oxodG in diabetes revealed its significant association with severe hyperglycemia, poor diabetes control, and severity of diabetic complications, as well as predictive value for diabetes-associated morbidity [34]. The relationship between u-8-oxodG and depression has been modestly investigated and provided controversial results, from an increase to no change, depending on the study-specific depression phenotype [35–37]. To our knowledge, this is the first study investigating u-8-oxodG in patients with diabetes and elevated depressive symptoms. While the results in our exercise group are in accord with previously reported reduction in u-8-oxodG elicited by moderate-intensity exercise in type 2 diabetes over 12 months [38], significant decrease in u-8-oxodG levels in psychoeducation and diabetes reeducation group is a novel finding, indicating that cognitive-behavioral approach might have beneficial effects on oxidative damage reduction in diabetic patients with subsyndromal depression. Additional support for this finding was a significant increase in serum uric acid level at follow-up.

Uric acid is an end-product of purine metabolism, with complex and multifarious roles in human physiology. Apart from causing gout, hyperuricemia has been identified as a risk factor for metabolic syndrome, type 2 diabetes, and cardiovascular diseases [39]. Recently proposed intriguing hypothesis linking hyperuricemia derived by an increased consumption of dietary fructose with an epidemic of cardiometabolic diseases attracted a lot of attention and created a paradigm of uric acid as being an exclusively harmful metabolite [40]. By contrast, uric acid has long been recognized as one of the most powerful endogenous antioxidants [41] and an efficient mediator of neuroprotection, cognitive function, and intellectual performance [42], as well as the most pronounced determinant of total plasma antioxidant capacity in diabetic patients [43]. Thus, maintaining uric acid levels within physiological range appears to be more favorable than radical lowering in prevention of various morbidities. Based on recent epidemiological evidence, including cardiometabolic risk assessment, a new, gender-independent upper limit of reference range for uric acid has been proposed at the level of 360 *μ*mol/L [44]. The present study reveals a significant increase in uric acid levels at follow-up, but well below proposed cut-off above which negative effects of hyperuricemia might be expected. On the contrary, the observed uric acid increase in our study is rather suggestive of an improved antioxidant capacity elicited by behavioral interventions in type 2 diabetic patients with subsyndromal depression.

Beneficial effect of cognitive-behavioral intervention in reducing oxidative stress and inflammatory response has been reported in hemodialysis patients suffering from insomnia [45], but rather sparse depression-related studies mainly focused on the assessment of low-grade inflammation biomarkers. In a very recent randomized trial evaluating a newly developed cognitive-behavioral program (DIAMOS) in diabetic patients with subsyndromal depression, a set of pro- and anti-inflammatory biomarkers was analyzed at basal and one-year follow-up [20]. No change in hs-CRP, IL-1, and adiponectin was observed, whereas a significant decrease in interleukin-1 receptor antagonist (IL-1RA) was interpreted as an indirect index of alleviated IL-1-related damaging mechanisms, presumably involved in the pathogenesis of micro- and macrovascular complications of diabetes. Likewise, our study could not demonstrate any influence of the behavioral interventions on hs-CRP and adiponectin, but we found other inflammatory markers, that is, leukocyte count and total serum sialic acid (TSA), significantly reduced, with the latter being largely affected (*η* ^2^ = 0.32) by the interventions performed.

Sialic acids comprise a family of heterogenous monosaccharides abundantly present on the cell membranes, with complex and multiple biological roles in cellular signaling and immunity [46]. TSA is a validated inflammatory and atherogenic marker that has been proposed as a novel biomarker of cardiovascular disease [47]. Elevated levels of TSA in newly diagnosed type 2 diabetic patients have been linked to the risk for later development of macrovascular complications [28]. Interestingly, TSA has been identified as a superior predictive biomarker of metabolic syndrome over the abundantly used hs-CRP [48]. Nevertheless, despite the obvious advantages, TSA has been largely underutilized in the research on combined inflammatory-metabolic pathologies and it has not been studied in depression until this study. Our results identify TSA as a sensitive biomarker of inflammation degree in subsyndromally depressed diabetic patients, whereas neither of the established biomarkers of low-grade inflammation, hs-CRP and adiponectin, was able to reflect the anti-inflammatory effect of the performed interventions. Significant decrease in leukocyte count, however, clearly confirmed postintervention attenuation of the cell-mediated inflammation response in our patients, with an important notice that the presence of acute infection at the assessments was excluded by total leukocyte count < 10.0 × 10^9^/L or hs-CRP < 5.0 g/L.

The ability of psychological intervention to reduce both depressive symptoms and inflammation has been evidenced in depressed breast-cancer patients [49]. Furthermore, due to the insightful study-design, Thornton et al. were able to demonstrate a bidirectional mechanistic relationship between depressive symptoms and inflammation. The paradigmatic view of the low-grade inflammation as the inflicting mechanism for depression has been at least complemented, if not refuted, by their finding on the mechanistic role of depressive symptoms lowering in the reduction of inflammatory response [49]. This explanatory mechanism might also be translated to our results, particularly considering a lack of any effect of interventions towards hyperglycemia and obesity in our study.

It is well known that the intensity of oxidative damage and inflammation in diabetes is remarkably and positively associated with the severity of hyperglycemia [18]. However, the observed reduction in oxidative damage and low-grade inflammation in our study could not be attributed to this mechanism, since no significant changes from baseline to follow-up were found in HbA_1c_, clinically considered to reflect a fair glucoregulation (Table 3). Obesity-derived low-grade inflammation is another recognized harmful component in the pathogenesis of type 2 diabetes and its complications [50]. Obesity induces a disbalance between pro- and anti-inflammatory cytokines/adipokines, resulting in low-grade inflammation and related adverse health outcomes ranging from metabolic syndrome and diabetes to cardiovascular diseases and cancer, as well as depression [49–51]. Reduction in body weight is accompanied by beneficial metabolic and hormonal profiles and a reduced inflammation [52], with increased production of anti-inflammatory adipokine adiponectin suggested to be among the most relevant features [53]. Since our subjects, classified as overweight/obese [54], retained their BMI at follow-up and revealed no changes in adiponectin levels, it is likely that the observed effects of interventions on the reduction in depressive symptoms and oxidative/inflammatory biomarkers in this study depended on another mechanism(s).

Chronic stress, with deregulation of stress-response adaptive mechanisms, is considered to be a common feature of both diabetes and depression [55]. There is a growing body of evidence suggesting that metabolic inflammation, associated with obesity and diabetes, inflicts neuroinflammation which causes serious disturbances of the hypothalamic-pituitary-adrenal (HPA) axis function and, consequently, leads to impairment of adaptive stress-response [56]. Moreover, chronic stress exposure has recently been demonstrated as a promoting factor for accelerated oxidative damage, whereby a sustained activation of HPA-axis emerged again as the key mediator [57]. Neuroendocrine effects of behavioral treatment for depression have not been extensively studied, but the evidence so far suggests a hypothetical pathway that connects the nurturing effects derived from the patient-therapist “bond” with reestablishment of neuroendocrine homeostasis [58]. Moreover, positive effects of physical exercise on preventing potentially harmful stress-related metabolic and psychological consequences have been presumably mediated by the same neuroendocrine mechanisms [59]. Considering unchanged metabolic profile of our subjects and remarkable improvements in depressive symptoms and oxidative/inflammatory biomarkers following either behavioral intervention, it is tempting, if not plausible, to speculate on the involvement of neuroendocrine mechanism(s) in achieving this beneficial response. It should be noted that a lack of between-group differences in changes observed in our study might not be explained by a phenomenon known as “regression toward the mean,” or just a natural course of parameters. Previous findings on persistence of depressive symptoms in people with type 2 diabetes indicate that depression is not only persistent [60, 61], but also associated with an increased risk of aggravating over time [62]. On the other hand, oxidative damage to DNA is increasing in healthy subjects throughout time as a result of ageing process [33], while inflammatory response and ageing-associated damage to biomolecules are even more pronounced in diabetes, due to a long-term exposure to hyperglycemia and advanced-glycated end-products [34, 50].

The strengths of this study are in examining a highly prevalent population of type 2 patients with depressive symptoms but no clinical depression, using a randomized comparative design, employing structured behavioral interventions and analyzing clinically relevant changes of simple-to-measure oxidative/inflammatory biomarkers. Its limitation is the lack of treatment as usual control intervention, precluding the possibility to investigate the course and outcomes of subclinical depression without any treatment. Also, the study was carried out in only one center—a tertiary diabetes clinic—which does not allow concluding about its feasibility in other clinical settings. Per-protocol analyses are presented in the study results which were confirmed by the intention to treat analysis based on baseline-observation-carried-forward approach. A lack of estimation derived from multiple predictors may be considered a limitation of the study.

## 5. Conclusions

This study has demonstrated that improvement in depressive symptoms is accompanied with reduced oxidative damage and inflammatory response in type 2 diabetic patients with subsyndromal depression. Testing mediating effects has indicated that medium-term improvement in depressive symptoms predicts long-term-reduction in oxidative damage, while mediating factors for observed reduction in inflammatory response should be further investigated. Clinical benefits of treating subsyndromal depression were shown to be comparable in all three treatment arms.

Regardless of the mechanisms involved, our study clearly indicates that enhanced treatment as usual, defined as brief patient-centred diabetes reeducation, is not inferior to psychoeducation and exercise in providing beneficial outcomes in subsyndromally depressed type 2 diabetic patients. Simple behavioral interventions are capable not only of alleviating depressive symptoms, but also of reducing the intensity of damaging oxidative/inflammatory processes, thereby mitigating the risk of development of diabetic complications.

## Figures and Tables

**Table 1:** Programs' objectives, methods of delivery, and specific features.

Study arm	Objectives	Methods of delivery	Specific features
Psychoeducation	(i) Recognizing and understanding feelings (ii) Becoming aware of dysfunctional thinking patterns (iii) Improving mood by pleasant activities (iv) Improving mood by solving problems (v) Managing automatic negative thoughts (vi) Communicating assertively, providing support	(i) Six weekly group sessions lasting for 90 minutes (ii) Interactive learning in small groups (5–8 participants) (iii) Self-help manual (iv) Workbook with practical exercises	Learning and discussions supported by a structured PowerPoint presentation Topics focused on diabetes-related issues and concerns

Physical exercise	(i) Acquiring warm-up, flexibility, stretching, and strengthening exercises (ii) Learning about physical activity and: glycaemic control; cardiovascular health; energy expenditure; peripheral nerves; mood; diabetes self-management	(i) Six weekly group sessions lasting for 90 minutes (ii) Practicing exercises and exchanging experiences in small groups (iii) Written material reminding of practiced exercises	Learning and discussions supported by a structured PowerPoint presentation Exercise intensity measured by a heart rate monitor, maintained in a light-to-medium intensity range Blood glucose and blood pressure measured before and after sessions

Enhanced treatment as usual	(i) Discussing recent laboratory findings (ii) Addressing diabetes-related concerns	(i) One group session lasting for 90 minutes (ii) Patient-centered counselling in the group	Written self-help instructions to cope with mood difficulties

**Table 2:** Baseline characteristics of participants by the intervention groups (*n* = 209).

	Psychoeducation	Physical exercise	Enhanced treatment as usual	Sig.^**∗**^
*N*	74	66	69	
Age (yrs.)	57.7 (6.2)	58.5 (4.8)	58.2 (5.6)	0.648
Female (*n*)	40	37	36	0.867
Education (yrs.)	12.6 (2.4)	12.4 (2.3)	12.2 (2.5)	0.623
Professional status (% employed)	30	27	30	0.502
Economic status (% poor)	13	12	13	0.317
Family status (% married/cohabitating)	76	72	76	0.488
Insulin use (%)	32	29	32	0.695
OHA use (%)	56	57	53	0.664
Insulin + OHA use (%)	12	14	15	0.632
Statin use (%)	68	71	71	0.786
HbA_1c_ (%)	7.4 (1.2)	7.2 (1.1)	7.2 (1.1)	0.384
HbA_1c_ (mmol/mol)	58 (13)	56 (13)	55 (11)	0.442
Body mass index kg/m^2^	30.64 (4.54)	29.44 (4.67)	29.96 (4.39)	0.297
Depressive symptoms (CES-D score)	19.7 (9.1)	20.5 (8.6)	19.7 (8.7)	0.813

Data are means (SD) or percentages.

^*∗*^Sig.: significance.^*∗*^ One-way ANOVA or *χ* ^2^ as appropriate.

OHA = oral hypoglycemic agents.

**Table 3:** One-year changes of depressive symptoms, inflammatory- and prooxidative biomarkers.

Group scores	Baseline M (SD)	12-month follow-up M (SD)	Effects	*F*	Overall *P* ^*∗*^	*P* 0 to 12 mo.^*∗∗*^	*η* ^2^
Depressive symptoms							
A (*n* = 64)	19.7 (9.1)	16.7 (7.9)	Time	12.51	<0.001	**0.003**	**0.07**
B (*n* = 58)	19.8 (8.2)	18.1 (9.8)	Time × group	0.61	0.656		0.007
C (*n* = 57)	19.0 (8.6)	17.4 (9.4)					
Leukocytes (×10^9^/L)							
A (*n* = 63)	7.5 (1.7)	7.3 (1.7)	Time	12.61	<0.001	**<0.001**	**0.07**
B (*n* = 58)	7.5 (1.6)	7.1 (1.5)	Time × group	1.32	0.265		0.001
C (*n* = 57)	7.8 (2.2)	7.1 (2.0)					
CRP (mg/L)^#^							
A (*n* = 64)	0.28 (0.49)	0.25 (0.44)	Time	2.38	0.09	1.00	0.01
B (*n* = 57)	0.34 (0.46)	0.32 (0.41)	Time × group	0.87	0.48		0.01
C (*n* = 57)	0.35 (0.41)	0.24 (0.39)					
Sialic acid (mmol/L)							
A (*n* = 64)	2.1 (0.31)	1.9 (0.29)	Time	84.57	<0.001	**<0.001**	**0.32**
B (*n* = 58)	2.1 (0.31)	1.8 (0.22)	Time × group	3.40	0.01		0.04
C (*n* = 57)	2.1 (0.28)	1.8 (0.24)					
u-8-OxodG (*μ*g/mmol)							
A (*n* = 64)	1.1 (0.5)	0.9 (0.4)	Time	10.66	<0.001	**<0.001**	**0.06**
B (*n* = 57)	1.4 (0.8)	1.1 (0.6)	Time × group	1.09	0.36		0.01
C (*n* = 57)	1.2 (0.7)	1.0 (0.4)					
Uric acid (*μ*mol/L)							
A (*n* = 64)	291.7 (85.2)	310.3 (77.5)	Time	13.39	<0.001	**<0.001**	**0.07**
B (*n* = 58)	303.4 (91.9)	323.2 (93.1)	Time × group	0.25	0.91		0.003
C (*n* = 57)	290.4 (77.9)	314.5 (83.4)					
HbA_1c_ (%)							
A (*n* = 64)	7.4 (1.3)	7.2 (0.9)	Time	2.70	0.07	0.08	0.02
B (*n* = 58)	7.2 (1.0)	7.2 (1.0)	Time × group	0.92	0.45		0.01
C (*n* = 57)	7.1 (1.0)	7.0 (1.0)					
HbA_1c _(mmol/L)							
A (*n* = 64)	57.8 (13.6)	55.9 (10.2)	Time	3.31	0.05	1.00	0.02
B (*n* = 58)	54.8 (11.4)	55.7 (10.7)	Time × group	0.90	0.45		0.01
C (*n* = 57)	54.8 (10.7)	53.9 (10.0)					
Adiponectin (mg/L)							
A (*n* = 63)	7.5 (2.3)	7.1 (3.8)	Time	0.73	0.44	1.00	0.004
B (*n* = 58)	9.1 (6.1)	9.0 (6.7)	Time × group	0.55	0.65		0.006
C (*n* = 57)	8.0 (5.0)	8.1 (5.3)					
Body mass index (kg/m^2^)							
A (*n* = 54)	30.6 (4.7)	30.6 (5.0)	Time	1.72	0.19	0.11	0.01
B (*n* = 53)	29.2 (4.5)	29.0 (4.6)	Time × group	1.31	0.27		0.01
C (*n* = 52)	30.1 (4.1)	29.4 (4.0)					

^*∗*^Two-sided significance of differences across time and between the groups (repeated measures ANOVA).

^*∗∗*^Two-sided significance of differences between baseline and 12-month assessments.

^#^Log-transformed data.
